# *Bacillus subtilis* DSM29784 attenuates *Clostridium perfringens*-induced intestinal damage of broilers by modulating intestinal microbiota and the metabolome

**DOI:** 10.3389/fmicb.2023.1138903

**Published:** 2023-03-16

**Authors:** Yuanyuan Wang, Yibin Xu, Guangtian Cao, Xihong Zhou, Qian Wang, Aikun Fu, Xiuan Zhan

**Affiliations:** ^1^Key Laboratory of Molecular Animal Nutrition of the Ministry of Education, College of Animal Sciences, Institute of Feed Science, Zhejiang University, Hangzhou, China; ^2^China Jiliang University, Hangzhou, China; ^3^Institute of Subtropical Agriculture, Chinese Academy of Sciences, Changsha, China; ^4^Yancheng Biological Engineering Higher Vocational Technology School, Yancheng, China

**Keywords:** subclinical necrotic enteritis, probiotic, intestinal barrier, intestinal inflammation, gut microbiota, gut metabolome, broiler

## Abstract

Necrotic enteritis (NE), especially subclinical NE (SNE), without clinical symptoms, in chicks has become one of the most threatening problems to the poultry industry. Therefore, increasing attention has been focused on the research and application of effective probiotic strains as an alternative to antibiotics to prevent SNE in broilers. In the present study, we evaluated the effects of *Bacillus subtilis* DSM29784 (BS) on the prevention of subclinical necrotic enteritis (SNE) in broilers. A total of 480 1-day-old broiler chickens were randomly assigned to four dietary treatments, each with six replicates pens of twenty birds for 63 d. The negative (Ctr group) and positive (SNE group) groups were only fed a basal diet, while the two treatment groups received basal diets supplemented with BS (1 × 10^9^ colony-forming units BS/kg) (BS group) and 10mg/kg enramycin (ER group), respectively. On days 15, birds except those in the Ctr group were challenged with 20-fold dose coccidiosis vaccine, and then with 1 ml of C. perfringens (2 × 10^8^) at days 18 to 21 for SNE induction. BS, similar to ER, effectively attenuated CP-induced poor growth performance. Moreover, BS pretreatment increased villi height, claudin-1 expression, maltase activity, and immunoglobulin abundance, while decreasing lesional scores, as well as mucosal IFN-γ and TNF-α concentrations. In addition, BS pretreatment increased the relative abundance of beneficial bacteria and decreased that of pathogenic species; many lipid metabolites were enriched in the cecum of treated chickens. These results suggest that BS potentially provides active ingredients that may serve as an antibiotic substitute, effectively preventing SNE-induced growth decline by enhancing intestinal health in broilers.

## 1. Introduction

Necrotic enteritis (NE), a ubiquitous poultry disease, is a severe intestinal disease caused by *Clostridium perfringens* (CP). Birds with acute NE may experience sudden death, with up to 50% mortality (Caly et al., 2015). However, the more common form of NE is subclinical as it may persist in broiler flocks without overt clinical manifestation; hence, there is a general consensus that the subclinical form of NE (SNE) is more harmful than the clinical form (Olkowski et al., 2008). In addition, due to the large potential economic costs associated with SNE and the high risk of pathogen transfer to the food chain and public health concerns, industry experts perceive this problem as a major issue (Van Immerseel et al., 2004). Antibiotics have been used to prevent coccidiosis and NE for many decades (Diarra and Malouin, 2014), however, growing concerns about drug residues and antibiotic resistance, as well as their potential harmful effects on the homeostasis of gut microbiota, restrict their usage (Silva et al., 2009). Meanwhile, halting the administration of antibiotics causes the animals to be more susceptible to infections, such as NE, which has a significant negative impact on production yields (Gadde et al., 2017). Therefore, identifying an approach that may gradually replace antibiotics as an effective method to control disease infection in poultry, while maintaining good production yields and the health of the birds, is of high importance.

Probiotic application for SNE prevention is becoming a common method in the post-antibiotic era (Eeckhaut et al., 2016). Numerous studies have shown that live probiotic bacteria can support the host’s physiological and immunological development, improve disease resistance, compete with pathogens for nutrients and adhesion, produce metabolites that can directly inhibit bacterial diseases, and support the growth of potentially beneficial microbial organisms in the intestinal tract (Ducatelle et al., 2015; Elshaghabee et al., 2017). In the poultry industry, *Bacillus* has exhibited the greatest potential among feed probiotics due to its ability to produce spores that are resistant to the high temperatures used in modern production of pelleted poultry feed, as well as to the low pH, bile, and enzymes present in the upper digestive tract of chickens (Elshaghabee et al., 2017). *Bacillus subtilis* (BS) is a gram-positive aerobic bacterium that is widely used in the production of heterologous proteins (Earl et al., 2008). It secretes a variety of enzymes to degrade various substrates, enabling bacteria to survive in the changing environment. In addition, BS is an ideal multi-functional probiotic that can potentially prevent pathogen growth and promote nutrient absorption (Olmos et al., 2020). BS DSM29784 (referred to here as BS) not only improves the growth performance of turkeys, but also improves their intestinal health (Mohammadigheisar et al., 2019) as well as that of chickens (Rhayat et al., 2017; Neijat et al., 2019b). Specifically, a 1 × 10^9^ cfu /kg BS diet may optimize growth performance compared to other doses under farm conditions (Mohammadigheisar et al., 2019). Our team has previously reported that supplementing with probiotic BS can serve as an effective substitute for broiler antibiotics to reduce the feed conversion rate and improve gut health (Verdes et al., 2020). Owing to the ability of BS to inhibit CP growth *in vitro* (unpublished results), it is further speculated to be an effective feed additive to control SNE in broilers.

The animal intestinal microbiota plays a key role in the collection, storage, and consumption of energy obtained from the diet (Krajmalnik-Brown et al., 2012). These functions not only improve the health but can also increase the weight of the animal (Krajmalnik-Brown et al., 2012). Interestingly, FAO (2013) reported that probiotic application for animal nutrition might function as a gut ecosystem enhancer (Organization F. A, 2013). Moreover, the interaction between the gut microbiota and the immune system mediates long-term microbial colonization in the gut (Wandro et al., 2018). The microbiota can then interact directly with the immune system, or indirectly *via* release of metabolites that can be directly absorbed by immune cells and epithelial cells (Wikoff et al., 2009; Dodd et al., 2017). Therefore, metabolic activity is an important feature of the intestinal flora and a potential mechanism of host flora interaction (Zarrinpar et al., 2018). For instance, short chain fatty acids (SCFAs) produced by bacteria can affect the health and integrity of intestinal epithelia and immune cells (Willemsen et al., 2003; Chang et al., 2014; Kelly et al., 2015). Moreover, early exposure to microorganisms and their metabolites is a normal part of the development process, which has a significant, yet underexplored, impact on the immune system (Wandro et al., 2018).

Although few studies have used metabolites alongside bacterial community profiling to explore the effect of probiotics on preventing SNE development, the current study aimed to evaluate the effect of dietary supplementation of BS on SNE prevention in broilers. To this end, we systematically studied the protective role of BS in the gut immune response during SNE infection caused by the major pathogen CP, by combining broiler models and multiomics analyses. Moreover, we investigated whether oral supplementation with BS effectively prevents SNE-related pathogenesis, and performance damage, as has been demonstrated for antibacterial agents. Furthermore, we analyzed the cecal metabolome and microbiome profiles in SNE broilers and controls. Specifically, we investigated whether changes in metabolites and the composition of gut microbiota are associated with SNE infection.

## 2. Materials and methods

All procedures were carried out in accordance with the Chinese Animal Welfare Guidelines and approved by the Institutional Animal Care and Use Committee of Zhejiang University (Permission number: ZJU2019-480-12).

### 2.1. Bacterial strain preparation and experimental diets

The probiotic bacteria used in the present study was BS, which was provided by the Chinese Academy of Sciences. This strain was cultured in Luria-Bertani broth (Fisher Scientific, Ottawa, ON, Canada) and incubated at 37°C overnight in a shaking incubator at 180 rpm. CP type-A strain (China Veterinary Culture Collection Center, Being, China) was used for infection in this present study. CP was cultured in a Reinforced Clostridial Medium (Huankai, Guangdong, China) in an anaerobic environment at 37°C for 24 h, and subsequently used for challenge. The two bacterial pellets were collected after incubation at 5,000 ×*g* for 10 min at 4°C, respectively. After washing twice with sterile phosphate buffer saline (pH 7.3), the prepared *Bacillus* powder (2 × 10^9^ cfu/g) was diluted with starch and added to the basic feed to a final concentration of 10^9^ cfu/kg. The same amount of starch was added to compensate for the differences in dietary nutrients for each group. The coccidiosis quadrivalent live vaccine for chickens was purchased from Foshan Zhengdian Biotechnology Co., Ltd. (Guangdong, China) (Wang Y. et al., 2021).

### 2.2. BS extract antimicrobial activity

BS has a unique potential to secrete highly active bactericidal compounds. Therefore, the inhibitory effect of BS cell-free extract on CP was tested. Briefly, approximately 100 ml of liquid seed medium was inoculated with 1% freshly grown BS suspension. The inoculated seed medium was cultured in a shaking incubator at 180 rpm at 37°C for up to 24 h. After incubating for 24 h, the fermentation broth was centrifuged at 4000 ×*g* for 15 min, and the cell-free supernatant was further filtered through a 0.45-μm polysulfonate membrane filter. The filtered cell-free supernatant was considered to be the crude bactericidal extract, and the agar well diffusion assay was used to test against selected strains. Cultures were incubated overnight with CP (1 × 10^8^ cfu) on 150 ml tryptose-sulfite-cycloserine agar medium (Huankai, Guangdong, China). A sterile cork borer with a diameter of 10 mm was used to cut the agar well. Next, the cell-free supernatants (200 μl) were added to the wells in the plate and incubated overnight at 37°C (Xu et al., 2018). ER (100 μg/ml) and sterile Reinforced Clostridial Medium were used as positive and negative controls, respectively.

### 2.3. Experimental design and bird husbandry

A total of 480 Lingnan Yellow feathered-broilers with similar initial weights were randomly allotted to four groups with six replicates per group and 20 chicks per replicate (10 males and 10 females). All chicks were housed in 24 floor pens (2 m × 4 m) covered with fresh wood shavings. Fresh water and diet were provided *ad libitum*. The chicks were kept under a 2 l-1D light–dark cycle every day. Broilers in the negative (Ctr) and positive (SNE) control groups were fed the basal diet. Broilers in the BS group were fed a basal diet containing *Bacillus* concentration of 10^9^ cfu/kg. Broilers in the ER group were fed a basal diet containing 10 g/t of enramycin (ER; Schering-Plough, Shanghai, China). The temperature of the room was maintained at 33–35°C for the first 3 d and then reduced by 2–3°C per week to a final temperature of 25°C and 60–65% humidity. The experimental diet was designed according to the requirements of the National Research Council. The composition and nutritional level of the basic diet are shown in Table 1.

**Table 1:** Composition and nutrient level of the basal diet (% as fed basis).

Ingredients, %	Starter (1–21d)	Grower (22–42d)	Finisher (43–63d)
Corn	62.5	67.5	75
Soybean meal	31	23.5	14.5
CPM^**c**^	2	4	5
Soybean oil	0.5	1	1.5
NaCl	0.3	0.3	0.3
CaHPO_4_	1.2	1	0.8
Limestone	1.5	1.3	1.2
Zeolite	-	0.4	0.7
Premix^a^	1	1	1
Total	100	100	100
**Nutrient levels**^**b**^**(%)**			
ME (MJ/kg)	12.22	12.59	12.97
CP	21.09	19.16	16.07
Lys	1.09	0.99	0.87
Met	0.49	0.38	0.35
Met+Cys	0.87	0.73	0.65
Calcium	0.9	0.85	0.69
Total phosphorus	0.58	0.52	0.45

^a^The Premix provides per kg of diet: Fe 80 mg, Cu 8 mg, Zn 60 mg, Mn 80 mg, I 0.35 mg, Se 0.15 mg, VA 9,600 IU, VD3 1,500 IU, VE20mg, Vk3 1 mg, VB1 2.2 mg, VB2 4.2 mg, VB6 4.2 mg, VB12 0.012 mg, nicotinamide 42 mg, D-calcium pantothenate 12 mg, folic acid 1.0 mg, D-biotin 0.18 mg, choline, 800 mg. ^b^ME is a calculated value; other nutrient levels are measured values. ^c^CPM is corn gluten meal.

### 2.4. SNE broiler model

The SNE broiler model was established as previously described but with a small modification (Wang Y. et al., 2021). On day 15, the SNE-challenged groups, in addition to the negative control group, received a 20-fold dose coccidiosis vaccine per bird by oral gavage. Each bird in each group was then gavaged with 1 ml of CP (2 × 10^8^ cfu/ml) per day on days 18–21 (also, no food will be provided in the night before previous night during the period of 18–21 days). Meanwhile, the birds in the negative control group instead received equivalent sterile phosphate-buffered saline (PBS) on day 15, and days 18–21. All samples were collected on day 35.

### 2.5. Measurement of growth performance and sample collection

On days 1, 21, 42, and 63 of the experiment, birds were weighed per whole replicate. The variables of growth performance [final body weight (BW), average daily feed intake (ADFI), average daily gain (ADG), and feed:gain ratio (F:G)] were measured (Wang Y. et al., 2021). In detail, dead birds were recorded and weighed to adjust the estimates of gain, intake, and feed conversion ratios as appropriate. The average daily gain, average daily feed intake (ADFI), and feed:gain ratio (F:G) were calculated (Wang Y. et al., 2021).

Sample collection was performed in accordance with our previously described methods (Wang Y. et al., 2021). Before sample collection, all broilers were given sufficient water while no diet was provided for 12 h before analysis. On day 35, two birds (close to average BW) per replicate were selected and weighed. The right vein was punctured, and 10 ml blood was collected into a procoagulant vacuum tube and centrifuged (3,500 ×*g*, 10 min at 4°C). Pure serum samples were pipetted and transferred into 1.5-mL sterilized Eppendorf tubes, and stored at −80°C for further analysis. The chicks were then euthanized by a well-trained team. First, the small intestine from each bird was removed, opened, and subjected to lesion scoring, by the same trained personnel, according to previously described methods (Johnson and Reid, 1970). Next, a 0.5 cm sample of the jejunum wall was fixed in 2.5% glutaraldehyde (pH 7.4) and 4% paraformaldehyde, respectively. Additionally, the mucosa of 10 cm sections of the jejunum and duodenum, were gently scraped off and collected. The cecal contents were also collected and snap-frozen (Wang Y. et al., 2021).

### 2.6. DNA extraction, 16S rRNA sequencing, and microbial composition analysis

According to our previously described methods (Wang Y. et al., 2021), the microbial genome DNA was extracted from cecal content samples (TIANamp Stool DNA Kit DP328, TIANGEN, JP). The DNA extract was stored at 20°C until further analysis. The extracted DNA was quantified using a NanoDrop ND-1000 spectrophotometer (Thermo Fisher Scientific, Waltham, MA, United States) and agarose gel electrophoresis. Bacterial 16S rRNA gene sequences (V3–V4 region) were amplified using the Premix Ex Taq™ Hot Start Version (Takara, Dalian, China) and the following universal primers: 319F (5′-ACTCCTACGGGAGGCAGCAG-3′) and 806R (5′-GGACTACHVGGGTWTCTAAT-3′). Each polymerase chain reaction (PCR) mixture was prepared in a final volume of 50 μl containing 12.5 μl of the master mix, 1 μM of each primer, 50 ng of template DNA, and PCR-grade water. PCR reactions were performed using a gradient PCR instrument (L96G; LongGene, Hangzhou, China). MiSeq Illumina sequencing was further performed using the sequencing reaction (Illumina Inc., San Diego, CA, United States) for paired-end reads. The paired-end reads were then assembled and merged using FLASH and then assigned to each sample according to the unique barcodes. High-quality tags were clustered into operational taxonomic units (OTUs) using Usearch in QIIME software based on 97% sequence similarity, and these OTUs were further subjected to analysis using the Greengene database with the RDP algorithm. Alpha and beta diversity was assessed, and partial least squares discriminant analysis (PLS-DA), as well as the unweighted pair-group method with arithmetic mean (UPGMA) analysis were conducted using QIIME. Linear discriminant analysis (LDA) effect size (LEfSe) analyses were performed using the LEfSe tool (Wang K. et al., 2017). The associations between biomarker genera in the two groups and selected predictive functions were determined by Spearman’s correlation analysis (SPSS 23.0). The raw data from the high-throughput sequencing were deposited in the NCBI database^1^ with the BioProject ID PRJNA714475.

### 2.7. Untargeted metabolome profiling using gas chromatography–mass spectroscopy

According to our previously described methods (Wang Y. et al., 2021), frozen cecal digest (0.5 g) were lyophilized for 24 h and then transferred into 1 ml of polyethylene tubes. The digest was then mixed with 100 μl of methoxyamine hydrochloride in pyridine (20 mg/ml) and vortexed vigorously for 30 s. The sample was heated at 37°C for 90 min, after which 200 μl of a 1% trimethylchlorosilane solution of bis(trimethylsilyl)-trifluoroacetamide was added. The samples were heated at 70°C for 60 min and then kept at room temperature for 30 min. Subsequently, the samples were centrifuged at 10,000 ×*g* for 10 min at 4°C, and 100 μl of the supernatant of each sample was transferred into a GC vial. After adding 400–500 μl n-hexane, the samples were used for gas chromatography–mass spectroscopy (GC–MS) in the automatic sampling mode.

Each 1 μl sample was injected into the Agilent 6890A/5973C system equipped with a fused silica capillary column (30.0 m × 0.25 mm i.d.) packed with 0.25 μm HP-5MS. Helium was used as the carrier at a constant flow rate of 1.0 ml/min. Each 1 ml sample was injected into the device. The column temperature was maintained at 70°C for 2 min, increased to 200°C at a rate of 10°C/min, increased to 280°C at a rate of 5°C/min, and then maintained for 6 min. Mass detection was performed in the full scan mode, with a detection range of 50–650 (m/Z). GC–MS raw data files were converted into mzXML format and analyzed using the XCMS toolbox with the R statistical language (v3.4.1); post editing was performed using Excel 2010 software. The results were organized into a two-dimensional data matrix, including retention time (RT), mass charge ratio (MZ), sample amount, and peak intensity (Yuan et al., 2019).

The processed data were first subject to principal component analysis (PCA) using SIMCA 14.1 (Umetrics, Malmo, Sweden) after unit variance scaling to evaluate the similarities and differences between each sample. PLS-DA was then performed using the SIMCA-P software (version 12.0; Umetrics AB, Umeå, Sweden) for group classification and discrimination analysis. The heat map was generated using HemI (Heatmap Illustrator)^2^ (Deng et al., 2014), and metabolite classification was performed using ClassyFire^3^ (Djoumbou Feunang et al., 2016). The metabolite list for each comparison was separately subject to pathway analysis, which was performed using MetaboAnalyst^4^ (Chong et al., 2018) according to the Kyoto Encyclopedia of Genes and Genomes (KEGG) pathway database.^5^ Significantly varied pathways were identified with a cut-off *p* < 0.05.

### 2.8. Jejunum morphology and histomorphological measurements

The paraffin sections were subjected to hematoxylin and eosin (H&E) staining for histopathology analysis. Transmission electron microscopy and scanning electron microscopy was performed for the jejunal tissue according to our previous described protocols (Wang Y. et al., 2021). Morphometric measurements of jejunum villi were performed according to a previous described method (Awad et al., 2009).

### 2.9. Total RNA extraction and quantitative real-time PCR

According to previously described methods (Wang Y. Y. et al., 2021), total RNA was extracted from powdered frozen intestinal mucosa (RNAiso Plus reagent, TAKARA, Tokyo, Japan) and reverse-transcribed using M-MLV reverse transcriptase (Takara Bio). Real-Time PCR was performed using SYBR^®^ Green Premix Ex Taq™ (Takara) and the ABI 7500 Fast Real-Time PCR system (Applied Biosystems, Carlsbad, CA, United States). The primers used are shown in Table 2. Results were normalized to the abundance of β-actin transcripts and relative quantification was calculated using the 2^−ΔΔCT^ method.

**Table 2:** Sequences of real-time PCR primers.

Gene name	Primers (5′-3′)	Products	GenBank
*Claudin-1*	F: TGGCCACGTCATGGTATGG	62	NM_001013611
	R: AACGGGTGTGAAAGGGTCATAG		
*Occluding*	F: GAGCCCAGACTACCAAAGCAA	68	NM_205128
	R: GCTTGATGTGGAAGAGCTTGTTG		
*Muc-2*	F: GCCTGCCCAGGAAATCAAG	59	NM_001318434
	R: CGACAAGTTTGCTGGCACAT		
*β -actin*	F: GAGAAATTGTGCGTGACATCA	152	NM_205518
	R: CCTGAACCTCTCATTGCCA		

Muc-2: Mucin-2.

### 2.10. Biochemical determinations

The activities of sucrase, amylase, and maltase in the duodenal mucosa were measured through colorimetric methods with a spectrophotometer. The assays were conducted using assay kits according to the manufacturer’s instructions (Nanjing Jiancheng Bioengineering, Nanjing, China). The absorbance was measured using an Infinite M200 Pro NanoQuant.

### 2.11. Enzyme linked immunosorbent assay

The levels of interleukin (IL)-1β (No. H002), IL-6 (No. H007), secretory immunoglobulin A (sIgA), interferon-gamma (IFN-γ; H052), tumor necrosis factor α (TNF-α; No. H052-1), and immunoglobulin G (IgG) were determined colorimetrically using ELISA kits (Nanjing Jiancheng Institute of Bioengineering), according to the manufacturer’s instructions.

### 2.12. Immunofluorescence staining

Staining was performed in three independent replicates to confirm the results. Tissue sections were deparaffinized in xylene, rehydrated with a series of graded ethanol, and washed in distilled water and PBS. The tissue sections were subsequently placed in a repair box filled with EDTA antigen repair buffer (ph8.0) and then repaired in a microwave oven. After natural cooling and washing with PBS, 3% bovine serum albumin (Solarbio, Beijing, China) was added to evenly immerse the tissue, which was then at 37°C for 30 min. After removal of the sealing liquid, tissue sections were incubated with IgA (goat polyclonal, working dilution 1:500; ab112814; Abcam, Cambridge, United Kingdom) antibodies at 4°C overnight. After being washed in PBS, sections were exposed to secondary antibody goat anti-rabbit IgG [H + L] (Jackson ImmunoResearch, West Grove, PA, United States; 111-545-003) at 37°C for 1 h. Finally, the sections were stained with 4′,6-diamidino-2-phenylindole (DAPI) solution (Servicebio, Wuhan, China; G1012) for 10 min under dark conditions at room temperature, and a Nikon Eclipse TI-SR fluorescence microscope and Nikon DS-U3 imaging system were used to analyze the samples.

### 2.13. Statistical analyses

The metabolic profile data were processed using the SIMCA software (version 13.0; Umetrics ab). PCA, projections to PLS-DA, and orthogonal PLS-DA were used to process the cecum metabolomic data. The effect of variables was assessed with the projection (VIP > 1) and Welch’s *t*-test (*p* < 0.05) values to obtain the profile of each metabolite.

In addition, other data were subjected to one-way analysis of variance in SPSS (version 22.0; IBM Corp., Armonk, NY, United States) and expressed as the mean ± standard error of the mean (SEM). Analyses of 16S rRNA gene sequencing data were conducted using the Benjamini & Hochberg -based algorithm to correct the *p* value to reduce the false positive rate. Differences between treatment means were examined using Tukey’s multiple range test. Statistical significance was set at *p* < 0.05.

## 3. Results

### 3.1. BS supplementation prevents SNE-induced growth decline in broilers

As shown in Table 3, higher F:G and mortality were observed in SNE on days 1–21 (*p* < 0.05) compared to those of the Ctr group, whereas no significant differences were observed between BS and ER treatments. On days 22–63, the SNE group presented lower BW and ADG than those of the other three groups (*p* < 0.05). Lower ADFI and higher mortality were found in the SNE group (*p* < 0.05) compared to those of the Ctr group, while no significant difference was observed between the BS and ER groups. The Ctr and ER groups showed significantly lower F:G compared with that of the SNE treatment group (*p* < 0.05), whereas no significant differences were found between SNE and BS groups. No significant differences were observed in overall F:G among all groups, whereas significantly lower ADG, ADFI, and higher mortality were observed in the SNE group compared with those in the other three groups on days 1–63 (*p* < 0.05).

**Table 3:** Effects of *Bacillus subtilis* DSM29784 treatment group on the growth performance of broilers.

Items	Ctr	SNE	BS	ER	SEM	*p*-Value
**1 ~ 21d**						
BW(g)	551.97	526.47	535.92	547.04	12.664	0.214
ADG(g/d)	24.14	22.95	23.38	23.92	0.607	0.225
ADFI(g/d)	41.99	42.34	42.06	42.22	1.146	0.99
F:G	1.74^b^	1.85^a^	1.80^ab^	1.77^ab^	0.037	0.057
Mortality (%)	1.67^b^	7.50^a^	5.00^ab^	2.50^b^	0.013	0.001
**22 ~ 63d**						
BW (g)	2676.99^a^	2215.68^c^	2508.00^b^	2600.14^a^	32.366	<0.001
ADG (g/d)	50.60^a^	40.22^c^	46.95^b^	48.88^ab^	0.725	<0.001
ADFI (g/d)	140.53^a^	124.18^b^	136.29^ab^	135.49^ab^	4.843	0.019
F:G	2.78^b^	3.09^a^	2.90^ab^	2.77^b^	0.096	0.012
Mortality (%)	3.38^b^	8.97^a^	6.14^ab^	4.21^ab^	0.019	0.039
**1 ~ 63d**						
ADG (g/d)	41.78^a^	34.46^c^	39.10^b^	40.56^a^	0.515	<0.001
ADFI (g/d)	102.09^a^	89.92^b^	98.88^a^	100.27^a^	2.822	0.002
F:G	2.44	2.61	2.53	2.47	0.064	0.082
Mortality (%)	5.00^c^	15.83^a^	10.83^b^	6.67^bc^	0.017	<0.001

ADG, average daily gain; ADFI, average daily feed intake; F:G, feed:gain ratio. ^a,b,c^Mean values within the same row with unlike superscript letters were significantly different (*p* < 0·05). SEM = standard error of mean. Each value represents the mean ± SEM of 6 replicates (*n* = 6). Ctr group, basal diet in control group; SNE group, basal diet + SNE (20-fold dose coccidiosis vaccine) + 1 ml of *Clostridium perfringens* (2 × 10^8^ cfu/ml coinfection); BS group, basal diet +1 × 10^9^ colony-forming units (cfu)/kg BS diet + SNE; ER group, basal diet +10 mg/kg enramycin (ER) + SNE.

### 3.2. The BS fermentation supernatant directly inhibits *Clostridium perfringens* growth

**Figure 1:**
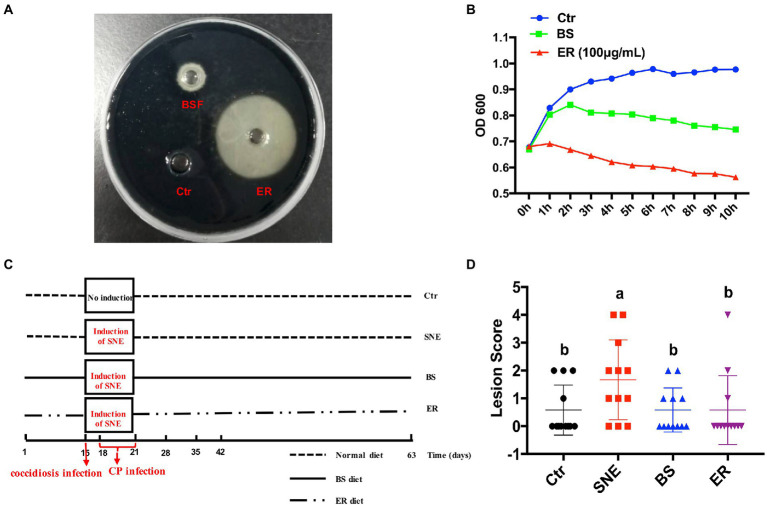
**(A)** *In vitro* antibacterial activity of BS fermentation supernatant (BSF) against *Clostridium perfringen* (CP). CP (1 × 10^8^ CFU/ml) were cultured in Trptose-sulfite-Cycloserine agar medium and treated with BSF, ER (100 μg/ml) and sterile Reinforced Clostridial Medium was used as a positive control and negative control, respectively, at 37°C. The size of the inhibition zone was observed **(B)** and analyzed statistically with a line chart. The OD600 kinetics were determined to analyze the effect of BSF against CP. **(C)** Schematic outline of the experimental design. SNE, subclinical necrotic enteritis. **(D)** Lesion scores of broilers. (a, b) Mean values with unlike letters between different groups are significantly different (*p* < 0·05). SEM, standard error of mean. Each value represents the mean ± SEM of 12 replicates (*n* = 12). The abbreviation of Ctr, SNE, BS and ER have the same meaning as Table 3. BSF, BS cell-free extract.

An *in vitro* bacterial inhibition assay was performed to assess the direct effect of BS fermentation supernatant on CP growth. BS had a significant inhibitory effect; however, the antibacterial effect, compared to that of the Ctr group, was not as strong as that elicited by 100 μg/ml ER (Figures 1A,B). The results show that BS fermentation supernatant has a direct effect on CP growth and proliferation *in vitro*.

### 3.3. BS supplementation attenuates intestinal lesions in broilers

We replicated the SNE model induced by the coccidiosis vaccine plus CP (Figure 1C). Intestinal lesions scored in the small intestine on day 35 are presented in Figure 1D. Birds in all treatments had low lesion scores, indicating that the challenge was subclinical. The SNE group had a higher lesion score than that of the other three groups (*p* < 0.05), and no significant difference was observed among the Ctr, BS, and ER groups (*p* > 0.05).

### 3.4. BS supplementation ameliorates SNE-induced intestinal mucosal injury

**Figure 2:**
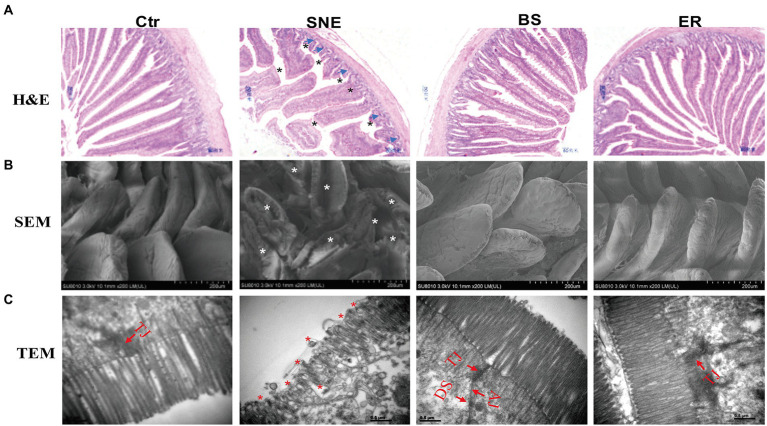
**(A)** Histopathology of the intestinal mucosa analyzed by H&E staining (top, scale bars = 80 μm). **(B)** Scanning electron micrograph (bottom, scale bars = 200 μm) and **(C)** transmission electron micrographs (bottom, scale bars = 0.5 μm) of jejunal brush border in broilers. TJ, tight junction; AJ, adherens junction; DS, desmosomes. Asterisk shows the pathology. Blue arrows show the features of necrotic cell death. The abbreviation of Ctr, SNE, BS and ER have the same meaning as Table 3.

H&E staining showed that the jejunum mucosa structure in the Ctr group was integrated, the intestinal villi were ordered, and the gland structure was clear and complete. However, the SNE treatment group showed an incomplete jejunum mucosa, with sparsely distributed villi of a relatively short length. With BS and ER pretreatments, the intestinal mucosal structure was significantly improved, and intestinal villi were higher (*p* < 0.05) with a denser arrangement (Figure 2A). Moreover, the Ctr group exhibited a higher villus height/crypt depth ratio than that of the SNE treatment group, as was also observed in the BS and ER groups (Table 4). We performed scanning electron microscopy (Figure 2B) and transmission electron microscopy (Figure 2C) to further examine the intestinal structure after the different treatments. The results showed that the Ctr group had complete jejunum villi, which formed full and closely arranged structures. The jejunum villi in the SNE group were severely damaged, whereas those in both the BS and ER groups showed greater improvement. These observations suggest that BS effectively prevented jejunal mucosal injury caused by SNE infection (*p* < 0.05).

**Table 4:** The jejunum histomorphology of broilers infected with *Clostridium perfringens*.

Items	Ctr	SNE	BS	ER	SEM	*p*-value
Villus height (μm)	1435.84^a^	1067.26^b^	1316.40^a^	1342.55^a^	45.258	<0.001
Crypt depth (μm)	194.94	188.85	179.06	182.24	9.100	0.434
Villus/Crypt ratio	7.53^a^	5.67^b^	7.39^a^	7.47^a^	0.401	<0.001

^a,b^Mean values within a row with no common superscript differ significantly (*P* < 0.05). SEM, standard error of mean. Each value represents the mean ± SEM of 15 replicates (*n* = 15). The abbreviation of Ctr, SNE, BS and ER have the same meaning as Table 3.

### 3.5. BS supplementation increases the expression of genes related to intestinal tight junctions

**Figure 3:**
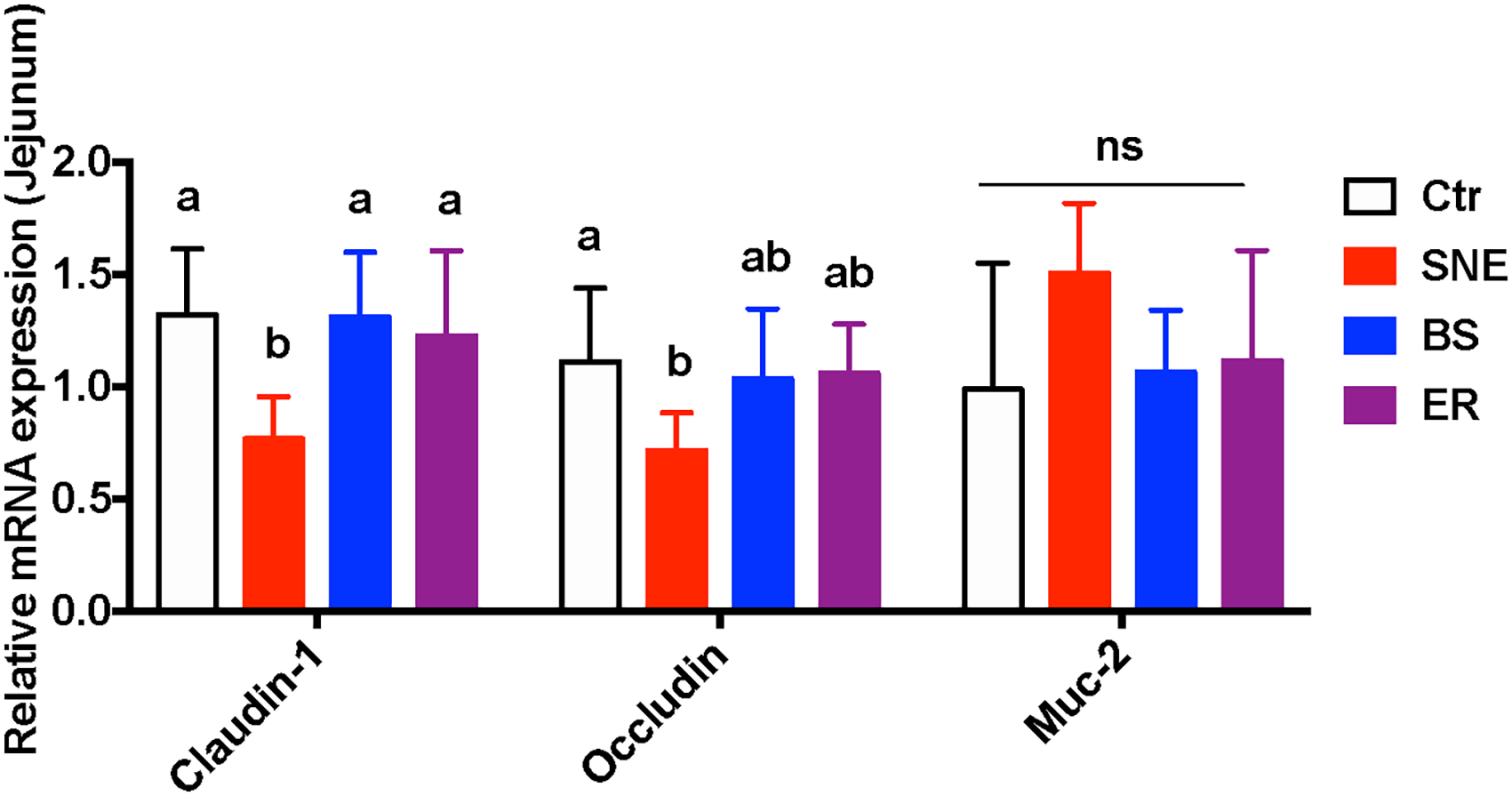
Changes on relative gene expression in the jejunum. (a, b) Mean values with unlike letters between different groups are significantly different (*p* < 0·05). ns, not significant; SEM, standard error of mean. Each value represents the mean ± SEM of 12 replicates (*n* = 12). The abbreviation of Ctr, SNE, BS and ER have the same meaning as Table 3.

As shown in Figure 3, compared with the Ctr group, the mRNA expression of *CLDN1* (claudin-1) and *OCLN* (occludin) in the jejunum of the SNE group were significantly reduced (*p* < 0.05). Meanwhile, BS pretreatment markedly upregulated the relative expression of *CLDN1* (70.95%, *p* < 0.05) and *OCLN* (44.47%, 0.05 < *p* < 0.1) in comparison with that in the SNE group. Notably, no significant differences in *CLDN1* or *OCLN* expression were observed among the Ctr, BS, and ER groups (*p* > 0.05). In addition, no significant difference was observed in the expression of *MUC2* among the treatment groups (*p* > 0.05).

### 3.6. BS supplementation alters the number of IgA^+^ B cells and immunoglobulins in the jejunum of broilers

**Figure 4:**
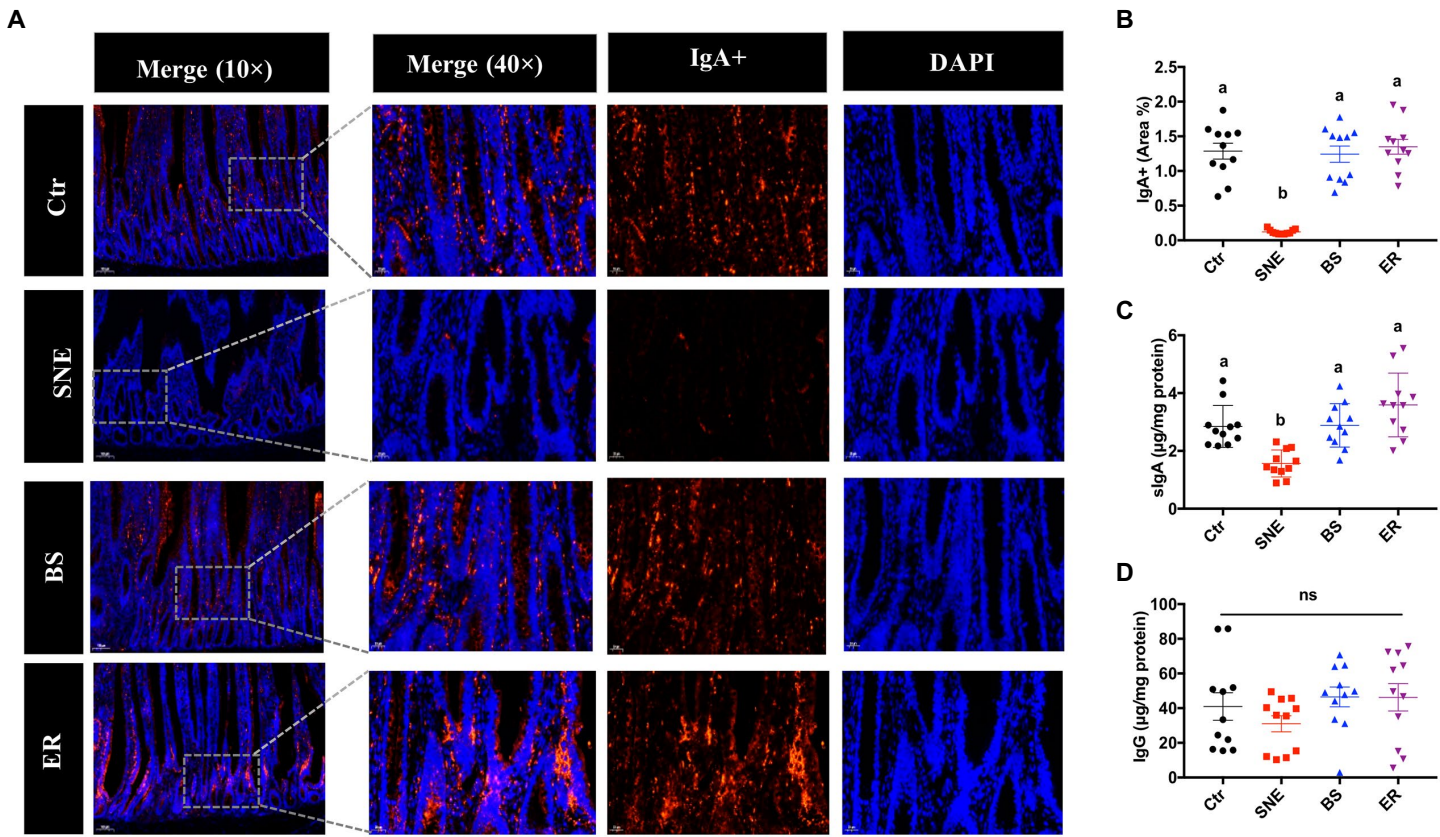
**(A)** IgA+ B cell abundance within the lamina propria in the jejunum under original magnification (×10 and ×40). **(B)** Relative quantification of the immunofluorescence results. **(C)** Changes in sIgA **(D)** and IgG levels in the jejunum. (a, b) Mean values with unlike letters between different groups are significantly different (*p* < 0·05). ns, not significant. SEM, standard error of mean. Each value represents the mean ± SEM of 12 replicates (*n* = 12). The abbreviation of Ctr, SNE, BS and ER have the same meaning as Table 3.

As shown in Figure 4, compared with the SNE group, the BS group had a higher number of IgA^+^ B cells in the lamina propria of the jejunum (*p* < 0.05), while no significant differences were observed among the Ctr, BS, and ER groups (*p* > 0.05). In addition, similar results were observed in the levels of sIgA in the jejunum. No difference was detected in IgG levels in the jejunum of the four experimental groups (*p* > 0.05).

### 3.7. BS supplementation alters digestive enzyme activity and immune response in broilers

**Figure 5:**
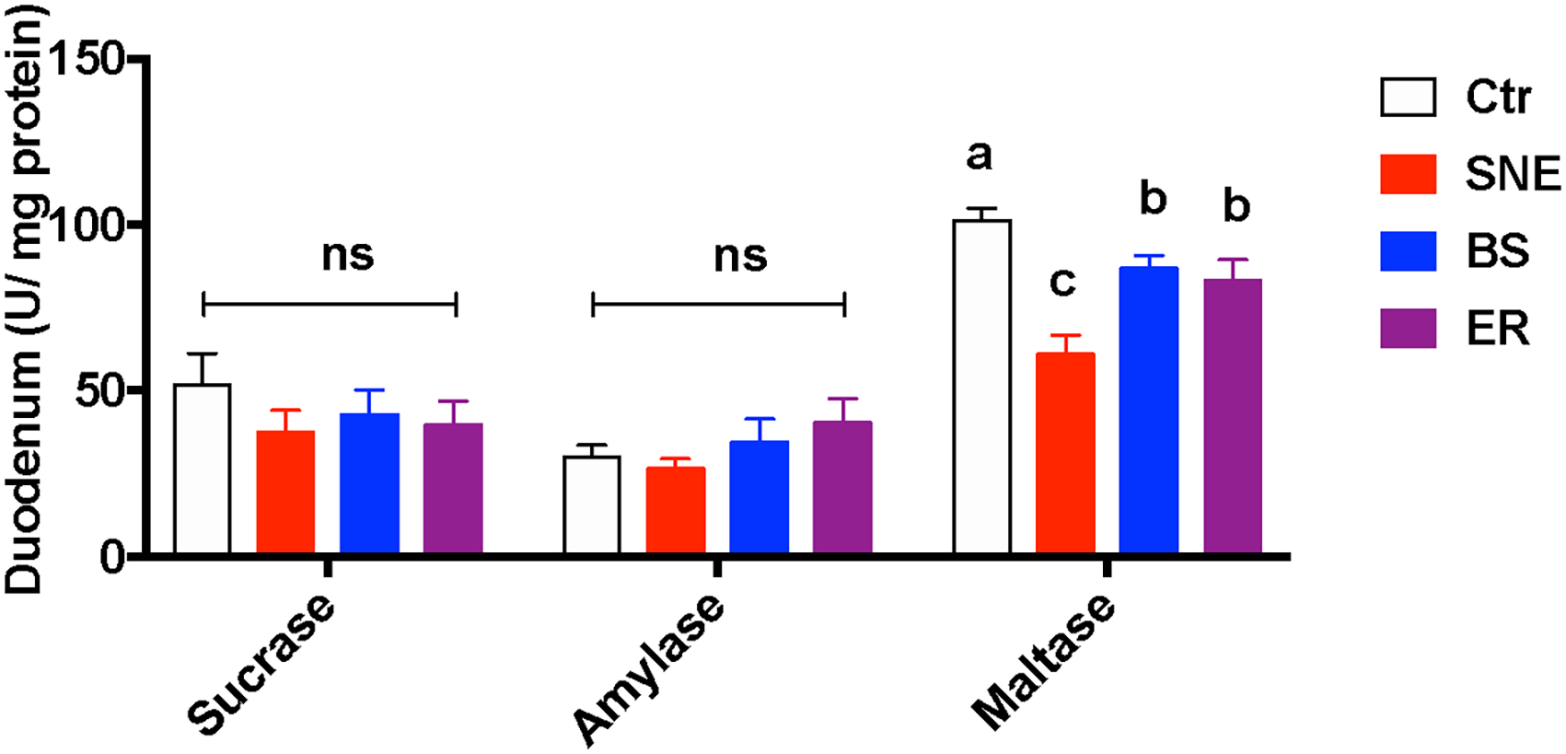
Duodenal mucosa biochemistry parameters of broiler chickens. Values are means (n = 10), with standard error of mean represented by vertical bars. (a, b, c) Mean values with unlike letters between different groups are significantly different (*p* < 0·05). SEM, standard error of mean. Each value represents the mean ± SEM of 12 replicates (*n* = 12). The abbreviation of Ctr, SNE, BS and ER have the same meaning as Table 3.

SNE significantly decreased maltase activity in the duodenum (*p* < 0.05) compared with that of the Ctr group. Furthermore, BS and ER markedly increased maltase activity compared to that in the SNE group (*p* < 0.05), whereas no difference was observed in the activities of sucrase and amylase (Figure 5). Cytokine secretion in the serum and jejunum mucosa is shown in Table 5. Proinflammatory cytokine secretion in the jejunum mucosa results revealed that the TNF-α level in SNE was markedly increased by 129.69 and 50.72% compared to that in Ctr and ER, respectively, while no significant differences were observed when compared to that of the BS group. In addition, the SNE group had higher serum levels of IFN-γ and TNF-α than the other three groups (*p* < 0.05), and no significant differences were observed between the BS and ER groups. No significant changes were observed in IL-1β or IL-6 levels in either the jejunum or serum.

**Table 5:** Effects of *Bacillus subtilis* DSM29784 treatment group on the cytokine secretion in serum and jejunum of broiler.

Items	Ctr	SNE	BS	ER	SEM	*p*-Value
**Jejunum**						
IL-1β (μg/mg protein)	1.50	2.49	1.39	1.58	0.427	0.055
IL-6 (μg/mg protein)	7.30	9.52	5.83	5.85	1.613	0.093
IFN-γ (μg/mg protein)	10.49	12.82	10.53	11.91	0.999	0.071
TNF-α (μg/mg protein)	2.29^c^	5.26^a^	3.96^ab^	3.49^bc^	0.603	<0.001
**Serum**						
IL-1β (ng/mL)	49.67	64.42	50.93	35.83	11.630	0.131
IL-6 (ng/mL)	51.59	64.32	60.66	55.96	9.098	0.534
IFN-γ (ng/mL)	112.60^b^	192.01^a^	114.48^b^	109.93^b^	22.298	0.002
TNF-α (ng/mL)	17.11^b^	37.05^a^	22.20^b^	18.63^b^	5.115	0.002

^a,b,c^Mean values with unlike letters between different groups were significantly different (*p* < 0·05). SEM = standard error of mean. Each value represents the mean ± SEM of 12 replicates (*n* = 12). The abbreviation of Ctr, SNE, BS and ER have the same meaning as Table 3.

### 3.8. BS supplementation induces a shift in the gut microbiota composition

**Figure 6:**
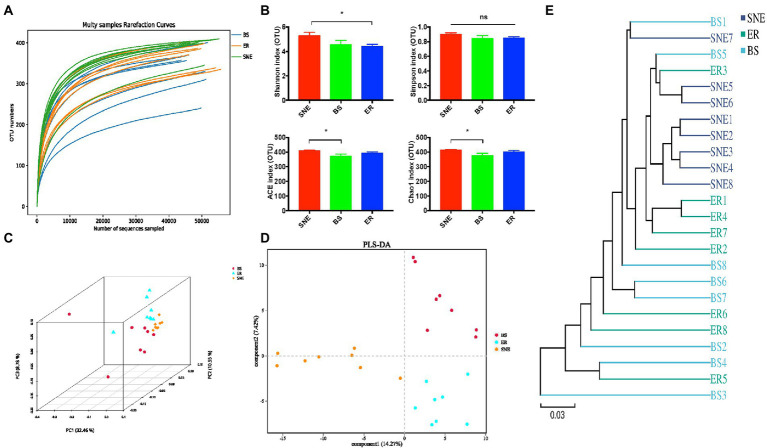
The cecal bacterial community of broilers among SNE, BS and ER treatments. **(A)** Rarefaction curve for total OTUs. **(B)** α-Diversity of gut microbiota was analyzed among SNE, BS and ER treatment groups by determination of principal dimension Simpson indices. **(C)** Three dimensional figures at the operational taxonomic unit (OTU) level obtained from PCoA based on the Bray–Curtis phylogenetic distance metric. **(D)** Partial least squares discriminant analysis of gut microbiota at the OTU level. **(E)** Unweighted pair-group method with arithmetic means (UPGMA) analysis based on the unweighted UniFrac. SEM, standard error of mean. Each value represents the mean ± SEM of 8 replicates (*n* = 8). The abbreviation of Ctr, SNE, BS and ER have the same meaning as Table 3.

Rarefaction curve analysis of OTUs in all samples approached the plateau (Figure 6A), indicating that the sampling depths were sufficient to capture the overall microbial diversity. Next, alpha diversity analysis was conducted using diversity indices (Shannon and Simpson) and richness estimates (Chao 1 and ACE). As can be seen in Figure 6B, the richness estimate increased significantly in the SNE group compared to that in the BS group (*p* < 0.05), whereas the diversity indices were similar among the three groups, except for the higher Shannon index in the SNE group than that in the ER group (*p* < 0.05). To examine the alteration in the composition of the gut microbiota, analysis of PCoA scatter plots was conducted and indicated a significant difference in the composition of the gut microbiota among the three groups (Figure 6C). The dissimilarity of the cecal microbiome presented was also confirmed by the separately clustered gut microbiota of the three groups shown in PLS-DA (Figure 6D) and UPGMA analysis (Figure 6E). In addition, the BS group was separated from the SNE group, and BS exhibited a tendency to cluster toward the ER group (Figures 6D,E), suggesting that BS administration attenuated the SNE-induced gut microbiota dysbiosis.

**Figure 7:**
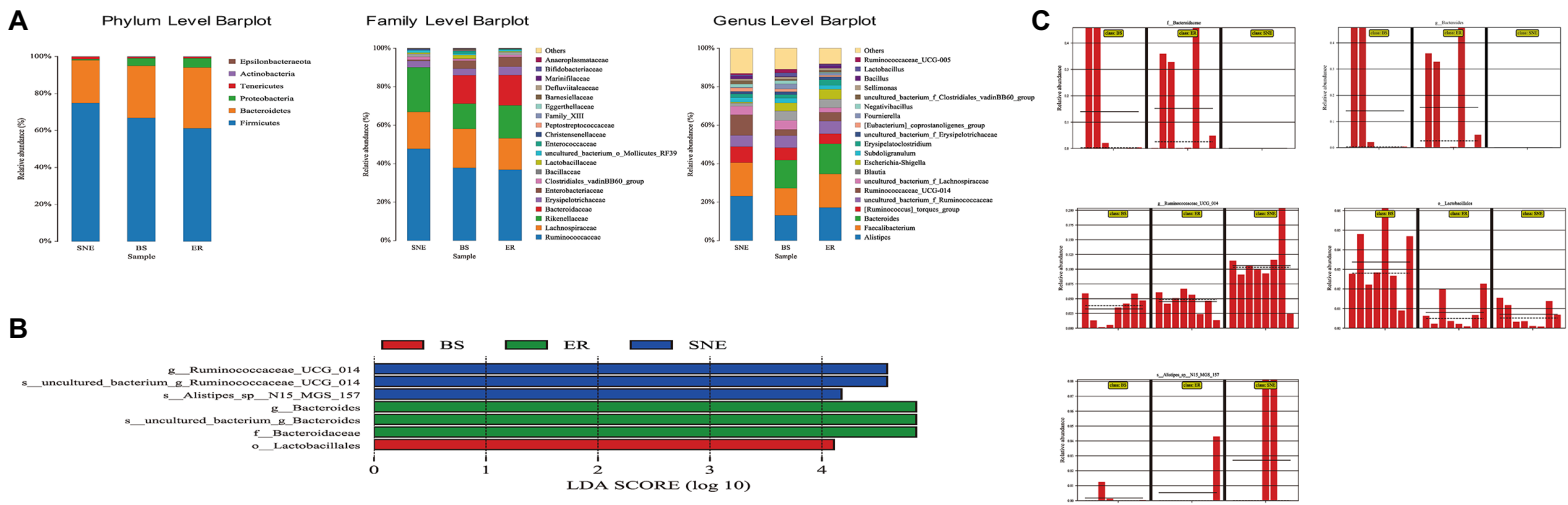
Structural changes in intestinal microbiota following dietary *Bacillus subtilis* DSM 29784 supplementation. **(A)** Relative abundance of microbial community in the cecum at the phylum, family, and genus levels. **(B)** LEfSe score plot of the discriminative microbial taxa (LDA score >4) that are more enriched in the BS (red), ER (green) and SNE (blue) groups. **(C)** Resulting bar plots display relative abundances of the phyla that are significantly altered, obtained from LEfSe analysis [o: order, f: family level, g: genus, s, species]. SEM, standard error of mean. Each value represents the mean ± SEM of 8 replicates (*n* = 8). The abbreviation of Ctr, SNE, BS and ER have the same meaning as Table 3.

Next, the relative microbial taxa abundances were compared among the three groups using analysis of variance. The top 20 most abundant microbial taxa at the phylum, family, and genus levels are shown in Figure 7A. At the phylum level, the abundance of *Bacteroidetes*, *Proteobacteria*, *Actinobacteria*, and *Epsilonbacteraeota* was increased, while that of *Firmicutes* and *Tenericutes* was decreased (*p* < 0.05) in the BS group compared with the SNE group. The ratio of *Bacteroidetes* to *Firmicutes* was higher in the BS (0.585) and ER (0.589) groups than in the SNE (0.324) group, although the difference was not significant, indicating that BS, similar to ER, profoundly benefited gut microbiota. At the family level, *Lactobacillaceae* (*p* < 0.05), *Enterococcaceae* (*p* < 0.05), and *Bifidobacteriaceae* (*p* < 0.05) were more abundant in the BS group than in the SNE group, whereas *Ruminococcaceae*, *uncultured_bacterium_o_Mollicutes_RF39* (*p* < 0.05), and *Christensenellaceae* (*p* < 0.05) were more abundant in the SNE group. At the genus level, the BS group had a higher abundance of *Lactobacillus* (*p* < 0.05) and a lower abundance of *Ruminococcaceae_UCG014* (*p* < 0.05) than that in the SNE group.

LEfSe was performed to explore the differences in bacterial content among the three groups (Figures 7B,C, LDA score >4). *Lactobacillales* were enriched in BS, but not in the other groups, suggesting that BS increased the number of beneficial bacteria. In addition, ER increased the abundance of *Bacteroidaceae* and *Bacteroides*, while the relative abundance of *Ruminococcaceae UCG 014* and *Alistipes* sp. *N15 MGS 157* increased in the SNE group.

### 3.9. BS supplementation alters cecal metabolic composition

**Figure 8:**
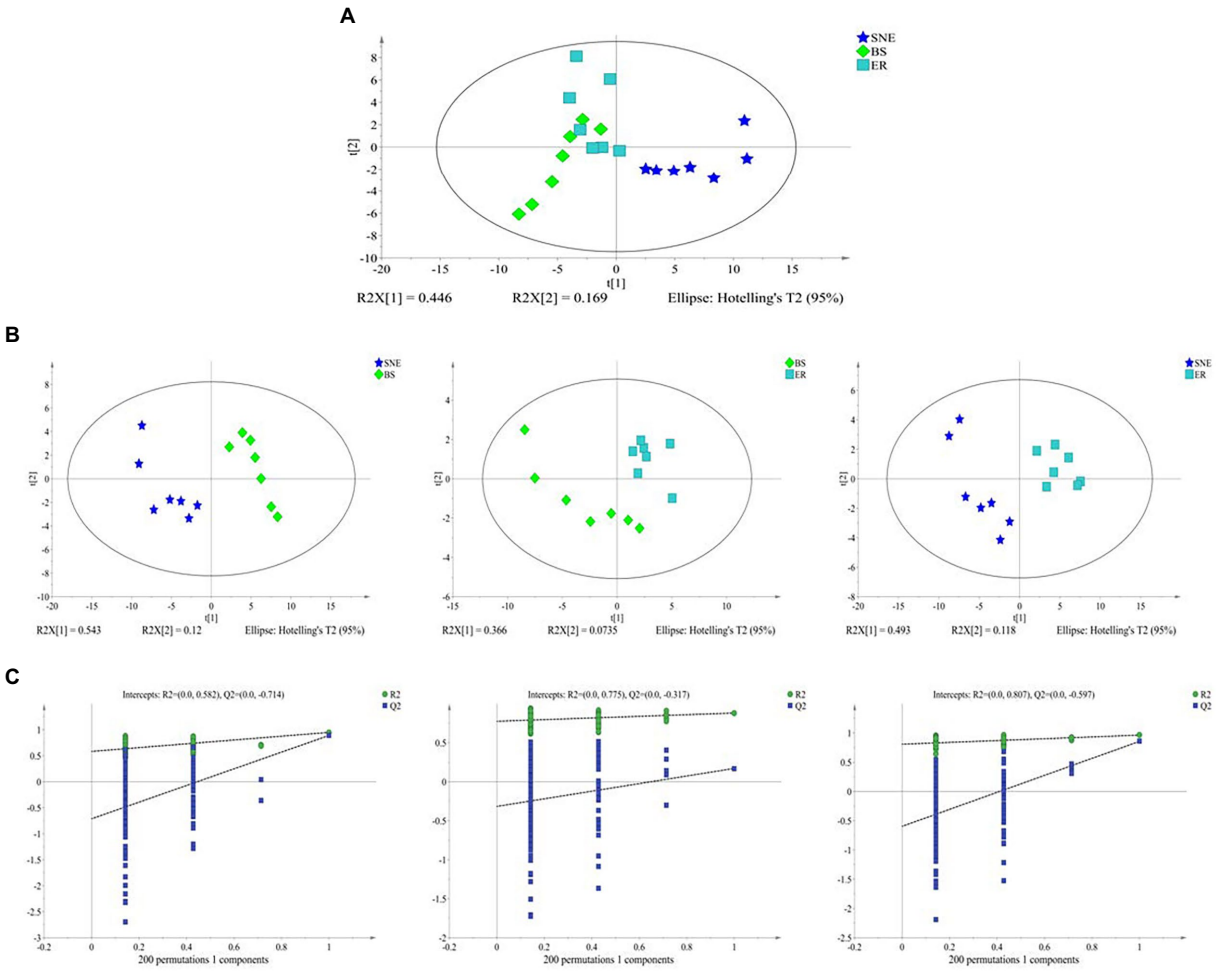
**(A)** Principal component analysis (PCA) score plots. **(B)** Projections to latent structure-discriminant analysis (PLS-DA) score plots of metabolic profiles obtained by BS vs. SNE [R2X = 0.663, R2Y = 0.949, Q2 = 0.894], ER vs. BS [R2X = 0.44, R2Y = 0.88, Q2 = 0.22] and ER vs. SNE [R2X = 0.795, R2Y = 0.991, Q2 = 0.898]; **(C)** permutation test of PLS-DA obtained by BS vs. SNE [left], ER vs. BS [middle] and ER vs. SNE [right]. SEM, standard error of mean. Each value represents the mean ± SEM of 7 replicates (*n* = 7). The abbreviation of Ctr, SNE, BS and ER have the same meaning as Table 3.

To explore the effect of BS supplementation on cecal microbiota, the cecal metabolite concentrations in the three groups were analyzed. Multivariate analysis between different groups was performed using PCA and PLS-DA. The results of the unsupervised PCA analysis indicated that the metabolome profiles of the three groups were separated from one another (R^2^X [1] = 0.446, R^2^X [2] = 0.169). However, the score plot also showed that the BS group clustered between the SNE and ER groups, with a tendency toward the ER group (Figure 8A). In addition, PLS-DA analysis was performed between the groups (BS vs. SNE, BS vs. ER, ER vs. SNE) (Figure 8B). Broilers in the SNE group compared to those in BS or ER groups were separated into distinct clusters according to their metabolic differences (BS vs. SNE: R2X = 0.663, R2Y = 0.949, Q2 = 0.894; ER vs. SNE: R2X = 0.795, R2Y = 0.991, Q2 = 0.898), while the BS group exhibited a tendency to cluster toward the ER group. Additionally, the PLS-DA permutation test demonstrated that the PLS-DA model was valid for the present study (Figure 8C). Overall, the results showed that the SNE model group had a metabolic composition distinct from that in the ER positive control group and the SNE model pretreated with BS.

**Figure 9:**
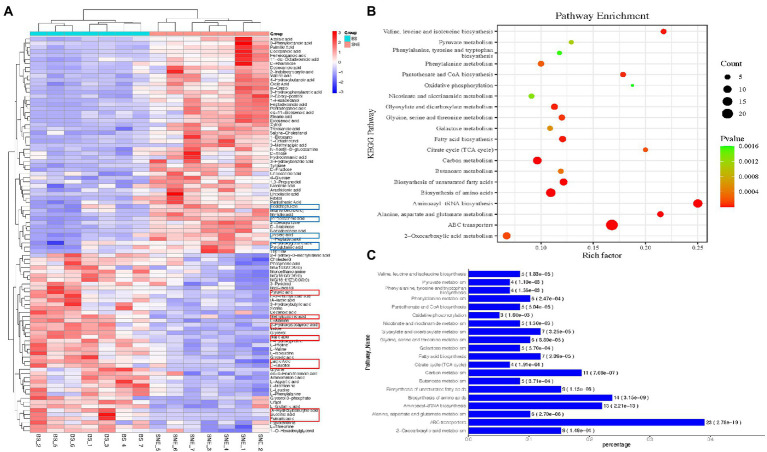
**(A)** Heat map of the significantly differentially-abundant metabolites between the BS group and the SNE group (VIP >1 and *p* < 0.05). A row presents the data obtained from a metabolite, and a column represents one sample. Red and green correspond to the increased and decreased levels of the metabolites, respectively; Bubble **(B)** and bar **(C)** charts of top 20 most enriched KEGG pathways. *p*-values are determined using two-tailed Student’s *t*-tests. SEM, standard error of mean. Each value represents the mean ± SEM of 7 replicates (*n* = 7). The abbreviation of SNE and BS have the same meaning as Table 3.

One of the primary aims of this study was to investigate the role of BS in the development of SNE. Subsequently, the metabolites that contributed to the change in the metabolic composition among the groups were selected based on the thresholds of VIP score >1 and *p* < 0.05. As depicted in Figure 9A, 97 differentially-abundant metabolites were identified from the comparison between the BS and SNE groups. In the BS group, 44 compounds (malic, lactic, pyruvic, and glyceric acids, among other compounds) were increased, and 53 compounds (2-hydroxyglutaric, linoleic, tetracosanoic, and nonadecanoic acids and tyrosine, among other compounds) were decreased in the BS group compared to those in SNE. Further metabolic pathway enrichment analysis demonstrated that broilers fed BS significantly altered their ABC transporters; carbon metabolism; and biosynthesis of aminoacyl-tRNA, amino acids, and unsaturated fatty acids, alanine, aspartate, glutamate, valine, leucine, and isoleucine; glyoxylate and dicarboxylate metabolism; pantothenate and CoA biosynthesis; and fatty acid biosynthesis (*p* < 0.00001, rich factor >0.15, Figures 9B,C).

### 3.10. Correlation between the cecal metabolome and gut microbiome

**Figure 10:**
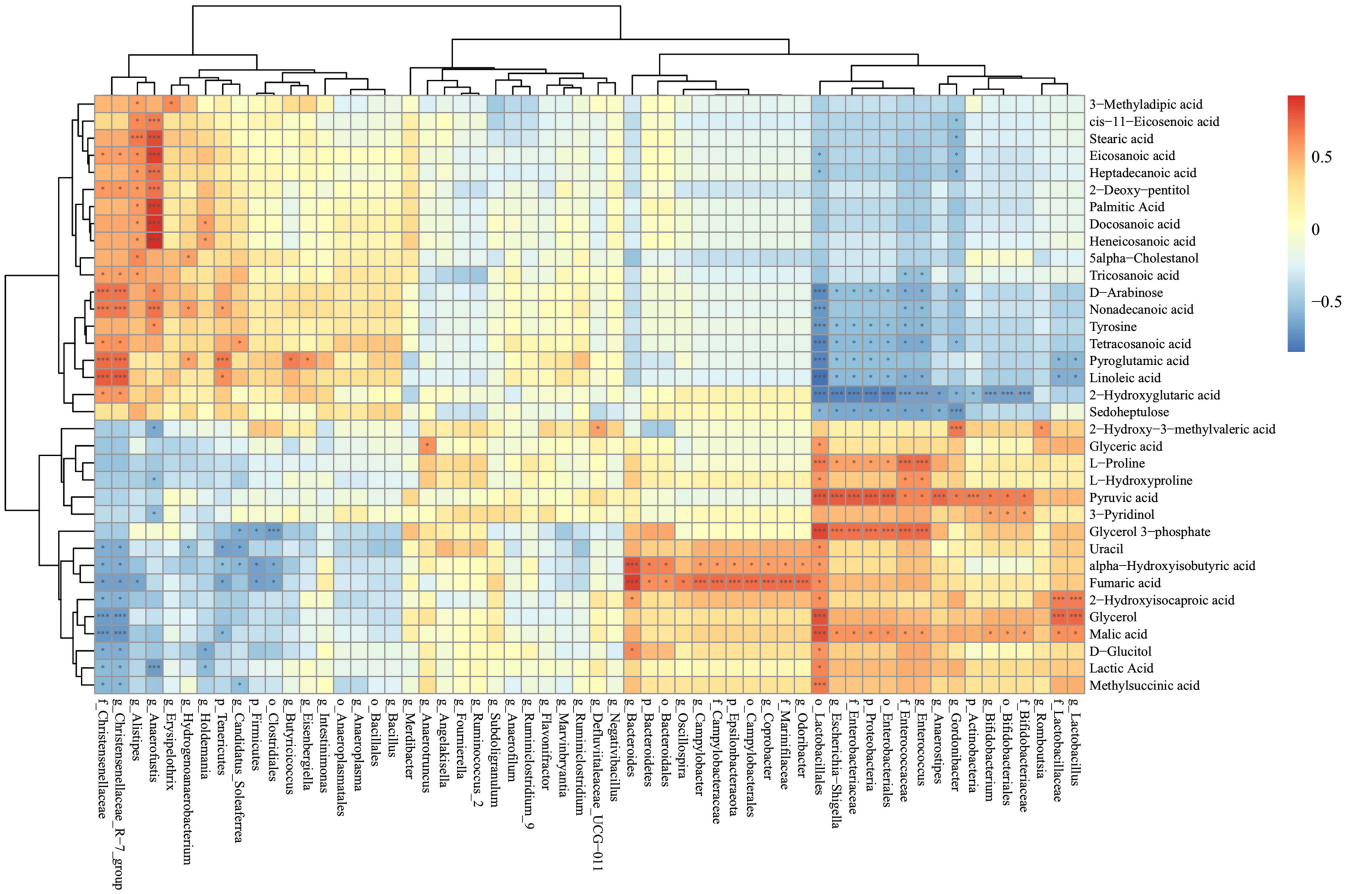
Correlations between significantly altered cecal metabolites and the bacterial strains between the BS-treated and SNE group. *p > 0.01 and *p* < 0.05, ***p < 0.01. SEM, standard error of mean. Each value represents the mean ± SEM of 7 replicates (*n* = 7).

To investigate the relationships between cecal metabolites and gut microbiota, Spearman correlation analysis was performed between cecal metabolites and cecal microbiota in SNE vs. BS (Figure 10). Beneficial bacteria such as *Lactobacillales* and *Bifidobacterium* were primarily correlated with metabolites that were higher in the cecum of the BS group, while they were negatively correlated with metabolites that were higher in the cecum of the SNE group. Specifically, the relative abundance of *Lactobacillales* was positively correlated with methylsuccinic, lactic, pyruvic, and malic acids, D-glucitol, glycerol, 2-hydroxyisocaproic, fumaric, and alpha-hydroxyisobutyric acids, uracil, glycerol 3-phosphate, L-hydroxyproline, L-proline, and glyceric acid. Meanwhile, *Lactobacillales* was negatively correlated with sedoheptulose, 2-hydroxyglutaric, linoleic, pyroglutamic, tetracosanoic, and nonadecanoic acids, tyrosine, and D-arabinose. The relative abundance of *Bifidobacterium* was positively correlated with malic and pyruvic acids, and 3-pyridinol, and negatively correlated with 2-hydroxyglutaric acid. Moreover, the relative abundance of *Alistipes* was negatively correlated with 3-methyladipic, cis-11-eicosenoic, stearic, eicosanoic, and heptadecanoic acids, 2-deoxy-pentitol, palmitic, docosanoic, and heneicosanoic acids, 5-alpha-cholestanol, and tricosanoic acid, and negatively correlated with fumaric acid. The relative abundance of *Christensenellaceae* and *Christensenellaceae_R-7_group* was positively correlated with methylsuccinic, lactic, and malic acids, D-glucitol, glycerol, 2-hydroxyisocaproic, fumaric, and alpha-hydroxyisobutyric acids, and uracil; meanwhile, they were negatively correlated with 2-hydroxyglutaric, linoleic, pyroglutamic, and tetracosanoic acids, tyrosine, nonadecanoic, tricosanoic, and eicosanoic acids, D-arabinose, and 2-deoxy-pentitol.

## 4. Discussion

Bacteriocins are ribosomally synthesized and are potent antimicrobial peptides mainly produced by *Bacillus* spp. (Khochamit et al., 2015). Historically, some *Bacillus* species have been considered safe for use in food and industry and as important vectors of ecological balance in animal models (Pedersen et al., 2002). Specifically, BS natto can inhibit pathogens such as *Salmonella typhimurium* and dysentery bacteria, which may be due to the production of bacitracin, polymyxin, 2,6-pyridinedicarboxylic acid, and other antibiotics (Zhang et al., 2020). Compared with other drugs, the antibacterial compound derived from BS natto has a broad antibacterial spectrum and is safe to the human body. Furthermore, previous studies have reported that BS inhibits the growth and virulence of *Staphylococcal* (Gonzalez et al., 2011; Piewngam et al., 2018). Similar results were obtained in the present study, and the antibacterial activity assay revealed that the BS fermentation supernatant directly inhibited CP growth. The findings of this study provide a theoretical basis for future *in vivo* animal experiments; however, the antimicrobial compounds require further investigation and characterization.

SNE is associated with huge economic losses owing to significantly worsened performance and intestinal necrosis, however, has a low associated mortality (Skinner et al., 2010). Previous statistical analyses showed that SNE is strongly correlated with feed conversion rate increase and growth retardation in broilers (Kaldhusdal and Hofshagen, 1992). Therefore, the use of feed additives to prevent SNE has been explored to reduce economic losses (Keerqin et al., 2021). In the animal industry, probiotics have been used to improve animal health and prevent various intestinal diseases, mostly since the prohibition of antibiotic growth promoters in animal husbandry (Mingmongkolchai and Panbangred, 2018). In this study, we found that dietary supplementation with BS led to higher BW and ADG in SNE-infected broilers. The growth-promoting effect of probiotics reported here is consistent with the results of other studies reporting the use of probiotics in broilers (Neijat et al., 2019a,b; Wang Y. Y. et al., 2021).

Nutrient absorption occurs primarily in the small intestine, particularly along the length of villi. Thus, the villi height directly determines the surface area absorbed by the intestine, which in turn affects the growth and development of animals (Salim et al., 2013). In contrast, shorter villi and deeper crypts may lead to decreased disease resistance and growth performance, malabsorption of nutrients, and increased gastrointestinal secretion (Mohammadagheri et al., 2016). As such, the villi length/crypt depth ratio is considered an important parameter for evaluating gut health and also implies that the epithelium is sufficiently mature and functionally active (Jayaraman et al., 2013). In the present study, BS effectively improved the morphology of the jejunum, including villi length and the villi length/crypt depth ratio, in SNE birds. Zhao et al. (2020) found that the addition of *Bacillus licheniformis* H2 increases the villi height: crypt depth ratio and villi height in the ileum (Zhao et al., 2020). We also showed that BS improves intestinal development and digestion primarily by increasing the villi height: crypt depth ratio in the jejunum (Wang Y. Y. et al., 2021). This evidence suggests that the well-developed small intestine may be related to the preventive effect of SNE.

Many luminal and systemic factors can independently influence barrier function and cause leakage of plasma proteins and watery diarrhea (Camilleri, 2019). For instance, tight junctions can facilitate paracellular permeability, which is an essential component of intestinal mucosal barriers (Kucharzik et al., 2001). These factors also comprise several unique proteins, including claudin-1, occludin, and Muc-2 (Wang et al., 2018). In this study, the relative abundance of jejunal transcripts of *CLDN1* and *OCLN* increased in BS and ER pretreatments compared with that in the SNE group. This result indicates that BS, similar to enramycin, could improve the intestinal physical barrier of SNE broilers.

Avian IgA exists in most intestinal cells, similar to mammalian IgA in mammals, and releases sIgA into the intestinal cavity through epithelial transport (Lindner et al., 2012). IgA protects the mucosal surface from viruses, toxins and bacteria by neutralizing or preventing the binding of pathogens to the mucosal surface (Lammers et al., 2010). Hence, sIgA determines the composition of the intestinal microbiota and affects the development of systemic immunity, thus, making it critical for the maintenance of mucosal homeostasis (Lammers et al., 2010). In the present study, SNE infection significantly reduced the number of IgA+ B cells and sIgA content, while BS pretreatment significantly improved, indicating that BS can enhance Igs to prevent SNE in broilers.

Inflammation is the main immune response of the body; however, an excessive immune response leads to a sharp rise in cytokines and production of the inflammatory cytokine storm, leading to immune system disorders and causing irreversible damage to host organs (Liu et al., 2020). In the present study, the levels of proinflammatory cytokines (IFN-γ and TNF-α) and chemokines (IL-1β and IL-6), which are key mediators in regulating the immune response, were measured. BS supplementation attenuated the concentration of IFN-γ in the jejunum and TNF-α in both the jejunum and serum. These results are in agreement with previous results of Wang H. S. et al. (2017) and Wang K. et al. (2017), who showed that feeding *Lactobacillus johnsonii* BS15 reduces the concentration of proinflammatory cytokines induced by SNE in the intestines of birds (Wang H. S. et al., 2017). The results indicated that dietary BS supplementation might reduce the adverse effects of inflammation on organs and maintain animal health.

In addition, the improvement of the intestinal epithelial integrity and mucosal immune function may further affect the intestinal microenvironment and improve nutrient digestibility (Jiang et al., 2009). Sucrase, maltase, and amylase play an important role in the fermentation process of related nutrients and ultimately affect the production performance and health of animals. In this study, BS was identified as a potential replacement for antibiotics in promoting maltase activity. Similar results were observed by Wang and Gu (2010), who reported that *Bacillus coagulans* may promote the growth of chickens and improve the digestibility of feed by secreting protease, α-amylase, xylanase, lipase, and other enzymes (Wang and Gu, 2010). Compared with SNE treatment, the increased maltase activities induced by BS and ER treatment could contribute to the higher ADG and BW in these treatments.

Maintenance of the host-microbiota balance is the key for homeostasis in animals (Wu et al., 2020). Changes in the microbiome and metabolome, as well as their interactions with the immune, endocrine, and mucosal systems, are related to a variety of diseases and vice versa. Diseases and pathological conditions often lead to an imbalance in intestinal microbiota and changes in microbial metabolites, leading to imbalances in metabolism and the immune system (Belkaid and Hand, 2014; Schmidt et al., 2018; Blacher et al., 2019). Nurmi and Rantala (1973) proposed that supplementation with probiotics might restore the protective gut microbiota and facilitate competitive exclusion (Nurmi and Rantala, 1973). To date, many studies have demonstrated the competitive exclusion of *Bacillus* in reducing the colonization of avian pathogens, such as reducing the counts of *Salmonella enteritidis* (La Ragione and Woodward, 2003), CP (Jayaraman et al., 2013; Jeong and Kim, 2014), *Enterobacteriaceae* (Jeong and Kim, 2014), *Campylobacter* (Guyard-Nicodème et al., 2016), and *Salmonella* (Vila et al., 2009; Knap et al., 2011) in the intestine. Moreover, in poultry nutrition, *Lactobacillus* and *Bifidobacterium* are generally considered beneficial as they promote intestinal health, improve the immune response of broiler systems, and enhance the health and performance of chickens (Salim et al., 2013). Alternatively, *Alistipes* spp. was recently identified as one of the top ten most abundant genera associated with human colorectal carcinoma (Feng et al., 2015). Moreover, Moschen et al. (2016) reported that facultative pathogenic *Alistipes* spp. induces colitis and site-specific tumors in IL10^−/−^ mice (Moschen et al., 2016). Kang et al. (2019) further reported that the abundance of *Alistipes* positively correlates with inflammatory genes, such as those coding for IL-6 (Kang et al., 2019). *Ruminococcaceae* are primairly responsible for fermenting dietary fiber and other plant components, such as inulin and cellulose, to produce SCFAs, which can be used as energy by the host (Scheppach and Weiler, 2004) and elicit anti-inflammatory effects in the intestine (Kles and Chang, 2006). In the present study, BS supplementation increased the abundance of *Lactobacillus* and *Bifidobacteriaceae*, while the abundance of *Ruminococcaceae* and *Alistipes* spp. was enhanced in the SNE group. The increased abundance of *Ruminococcaceae* found in the SNE group may be due to excessive inflammation in the intestine. Meanwhile, the increased abundance of beneficial bacteria in the BS group may have inhibited the growth of pathogens, such as *Erysipelotrichaceae* and *Escherichia-Shigella.* In summary, our results indicate that the addition of BS promotes the growth of beneficial bacteria while inhibiting the colonization of harmful bacteria to prevent the imbalance of intestinal flora and inflammatory injury caused by SNE.

A few studies have been performed on the metabolomic patterns in NE animal models. However, bacterial metabolites are essential elements in the interaction between the microbiota and the host (Russo et al., 2019). Metabolomics can identify different patterns of small molecules produced in the metabolic process of host and microbial cells, which may help to identify biomarkers of microbial patterns and disorders (Tuohy et al., 2009; Cevallos-Cevallos et al., 2011; Ponnusamy et al., 2011). The systemic effects of the gut microbiota are attributed to the less studied SCFAs, which are produced in the gut as the final products of fiber fermentation (Cook and Sellin, 1998). Volatile fatty acids, together with branched chain fatty acids, lactic acid, and other acids, play an important role in the gastrointestinal tract of birds by inhibiting the growth of various pathogenic bacteria (Svihus et al., 2013). This is achieved through the pH reduction caused by the acids, leading to inhibition of metabolic reactions, thereby reducing bacterial growth (Cherrington et al., 1991; Youssef et al., 2020). Therefore, high volatile fatty acids in the gut are generally considered healthy for the intestines (Timbermont et al., 2011). The current study showed that BS supplementation markedly increased the concentrations of lactic acid; hence, the production of acids may alleviate the intestinal mucosal damage caused by CP.

Increasing evidence shows that release of microbiota metabolites may affect the health of the host. In fact, recent studies have shown that the contribution of intestinal microbiota to host immune regulation is primarily due to microbial metabolism (Huttenhower et al., 2012; Li et al., 2014). Our results revealed that intestinal metabolites fluctuated with the structure of the intestinal microbiota. Pretreatment with BS regulated the levels of 97 metabolites, including benzenoids, lipids, nucleotides, organic acids, and organic nitrogen/oxygen compounds. These metabolites are primarily involved in several important metabolic pathways, including ABC transporters, carbon metabolism, as well as amino acid, fatty acid, and unsaturated fatty acid biosynthesis. Butyrate is an anti-inflammatory microbial metabolite that is important for intestinal homeostasis (Riviere et al., 2016). Moreover, butyric acid-producing bacteria coexist with *Bifidobacteria* (Riviere et al., 2016). This was also shown in our finding that malic acid, which is positively correlated with *Bifidobacteria,* was more abundant in the BS group. The results of the present study imply that many microorganisms may be involved in the alteration of intestinal metabolomics, thus affecting intestinal health. These findings indicate that the observed alternations in cecal metabolites upon coccidiosis vaccine plus CP coinfection were likely derived from the gut microbiota. Collectively, the strong correlations observed between the gut microbial changes and shifted metabolic levels indicated that SNE may have resulted in significant changes in the gut microbiota, leading to marked shifts in host metabolite abundance. These shifts result in the dysregulation of the host immune response leading to poorer growth properties in broilers.

In summary, we found that BS pretreatment significantly prevented the SNE-induced decrease in broiler growth performance. This may be because BS pretreatment increased villi height and maltase activity while decreasing the mucosal inflammatory response. In addition, BS pretreatment might modulate intestinal microbial composition and the gut metabolic profile as part of the microbial function. These findings provide a better understanding of the mechanism by which BS or ER promotes the prevention of SNE, which could provide useful insights for the development of an effective and safe alternative to antibiotics in the poultry industry.

## Data availability statement

The datasets presented in this study can be found in online repositories. The names of the repository/repositories and accession number(s) can be found in the article/supplementary material.

## Ethics statement

The animal study was reviewed and approved by all procedures were carried out in accordance with the Chinese Animal Welfare Guidelines and approved by the Institutional Animal Care and Use Committee of Zhejiang University (permission number: ZJU2019-480-12).

## Author contributions

XZha and YW conceived and designed the experiments. YW, QW, YX, XZho and AF performed the experiments. YW analyzed the data, made the figures, and wrote the paper. YW, XZha and GC revised the manuscript. All authors contributed to the article and approved the submitted version.

## Funding

This study was supported by the Key R&D program of Zhejiang Province (project: 2023C02026), China Agriculture Research System of MOF and MARA (project: CARS-41, Beijing, China), and the project of Hangzhou Agricultural and social development key research and development (project: 2022ZDSJ0157).

## Conflict of interest

The authors declare that the research was conducted in the absence of any commercial or financial relationships that could be construed as a potential conflict of interest.

## Publisher’s note

All claims expressed in this article are solely those of the authors and do not necessarily represent those of their affiliated organizations, or those of the publisher, the editors and the reviewers. Any product that may be evaluated in this article, or claim that may be made by its manufacturer, is not guaranteed or endorsed by the publisher.

## Footnotes

^1^https://www.ncbi.nlm.nih.gov/sra

^2^http://hemi.biocuckoo.org/

^3^http://classyfire.wishartlab.com/

^4^http://www.metaboanalyst.ca

^5^https://www.kegg.jp/

## Abbreviations

ADFI: Average daily feed intake
ADG: Average daily gain
BS: *Bacillus subtilis* DSM 29784
BW: Body weight
cfu: Colony-forming units
CP: *Clostridium perfringens*
ELISA: Enzyme-linked immunosorbent assay
ER: Enramycin
F:G: feed:gain ratio
H&E: Hematoxylin and eosin
IFN-γ: Interferon-gamma
IgG: Immunoglobulin G
IL: Interleukin
KEGG: Kyoto Encyclopedia of Genes and Genomes
LDA: Linear discriminant analysis
MUC2: mucin 2
NE: Necrotic enteritis
PBS: Phosphate-buffered saline
PCA: Principal component analysis
PCR: Polymerase chain reaction
SEM: Scanning electron microscopy
sIgA: secretory immunoglobulin A
SNE: Subclinical necrotic enteritis
SPSS: Statistical Product and Service Solutions
TEM: Transmission electron microscopy
TNF-α: Tumor necrosis factor α
VIP: Variable importance in projection
